# A single amino acid in the F_2_ subunit of respiratory syncytial virus fusion protein alters growth and fusogenicity

**DOI:** 10.1099/vir.0.055368-0

**Published:** 2013-12

**Authors:** Heather A. Lawlor, Jeanne H. Schickli, Roderick S. Tang

**Affiliations:** MedImmune, One MedImmune Way, Gaithersburg, MD 20878, USA

## Abstract

Respiratory syncytial virus (RSV) causes severe lower respiratory tract infection in children, especially in infants less than 1 year of age. There are currently no licensed vaccines against RSV. rA2ΔM2-2 is a promising live-attenuated vaccine candidate that is currently being evaluated in the clinic. Attenuation of rA2ΔM2-2 is achieved by a single deletion of the M2-2 gene, which disrupts the balance between viral transcription and replication. Whilst performing a manufacturing feasibility study in a serum-free adapted Vero cell line, differences in growth kinetics and cytopathic effect (CPE) were identified between two rA2ΔM2-2 vaccine candidates. Comparative sequence analysis identified four amino acid differences between the two vaccine viruses. Recombinant rA2ΔM2-2 viruses carrying each of the four amino acid differences identified a K66E mutation in the F_2_ fragment of the fusion (F) protein as the cause of the growth and CPE differences. Syncytium-formation experiments with RSV F protein carrying mutations at aa 66 suggested that a change in charge at this residue within the F_2_ fragment can have a significant impact on fusion.

## Introduction

Respiratory syncytial virus (RSV) is an enveloped, single-stranded, negative-sense RNA virus of the family *Paramyxoviridae*, subfamily *Pneumovirinae*. RSV causes lower respiratory tract infection in children, with a high incidence of serious disease in infants less than 1 year old (Nair *et al.*, 2010). Although prophylaxis with palivizumab antibody is available for high-risk infants, there are currently no licensed vaccines to prevent severe RSV disease (Johnson *et al.*, 1997). A variety of approaches to RSV vaccination have been evaluated over the years including subunits, virus-like particles and live-attenuated vaccines (Collins & Melero, 2011; Schickli *et al.*, 2009). The use of a non-live RSV vaccine for naïve infants is problematic because formalin-inactivated RSV, the first and only non-live RSV vaccine to be tested in naïve infants, not only was ineffective but the vaccinees also experienced a more severe disease upon subsequent reinfection with RSV than unvaccinated children. This phenomenon has been termed RSV enhanced disease (Kapikian *et al.*, 1969; Kim *et al.*, 1969). In contrast, live-attenuated vaccines are promising, and have been evaluated extensively in RSV-naïve children and infants in the clinic. None of the live-attenuated RSV vaccine candidates tested to date has been shown to cause enhanced disease in RSV-naïve infants or children (Karron *et al.*, 2005; Wright *et al.*, 2007). From an immunological perspective, live-attenuated virus is expected to mimic most closely the natural route of infection and, in turn, stimulate protective mucosal, humoral and cellular immune responses without the need for adjuvants.

One of the major challenges in developing a safe and effective live-attenuated RSV vaccine is maintaining the delicate balance between limiting virus replication in the host and delivering a high enough antigen load to induce a protective immune response. Most live-attenuated RSV vaccine candidates studied to date rely on point mutations to attenuate growth. Reversion of these point mutations can result in partial reversion of the attenuation phenotype as was observed in the rA2cp248/404/1030ΔSH clinical trial (Karron *et al.*, 2005). Partial reversion raises concerns about transmission of less-attenuated virus to vulnerable contacts of vaccinees.

Potential reversion of the attenuation phenotype can be mitigated by employing gene deletion strategies such as that in rA2ΔM2-2, a promising live-attenuated vaccine candidate. Attenuation is achieved by a deletion in the RSV genome that eliminates expression of the M2-2 gene (Cheng *et al.*, 2001; Jin *et al.*, 2000, 2003; Teng *et al.*, 2000), the product of which is thought to regulate the switch from transcription of viral mRNA to replication of the viral genome (Bermingham & Collins, 1999). Although rA2ΔM2-2 virus can grow to high titres in Vero cells, it is attenuated in mice, cotton rats and non-human primates (Cheng *et al.*, 2001; Jin *et al.*, 2000, 2003; Teng *et al.*, 2000). The advantages that distinguish rA2ΔM2-2 vaccine candidates from other live-attenuated RSV viruses are: (i) an attenuation phenotype that results from a gene deletion, which is less likely to revert than point mutations, and (ii) attenuated growth that does not highly compromise the expression level of viral antigens, thereby helping to maintain a high level of antigen load.

As part of a manufacturing feasibility assessment, two versions of rA2ΔM2-2, designated here as rA2ΔM2-2(MEDI) and rA2ΔM2-2(NIH), were evaluated in a serum-free (SF) adapted Vero cell line previously used to manufacture clinical trial vaccines (Yuk *et al*., 2006; Kaur *et al.*, 2008). In this manufacturing SF Vero cell line, the two versions of rA2ΔM2-2 showed different growth kinetics and cytopathic effect (CPE). The growth differences were unexpected, because both versions are derived from the A2 strain of RSV and share >98 % sequence identity. Alignment of their genome sequences identified four predicted amino acid differences in three viral proteins: the non-structural protein NS2, the nucleoprotein (N) and the fusion protein (F). Each of the different amino acids found in rA2ΔM2-2(NIH) was introduced into rA2ΔM2-2(MEDI) in order to assess their effect on growth. In this way, we identified the amino acid at position 66 in the F_2_ fragment of the RSV F protein as the genetic determinant of the observed growth differences between the two rA2ΔM2-2 viruses. Substitution of different amino acids at position 66 of RSV F further demonstrated that basic amino acids with positively charged side chains promote fusion activity and growth of rA2ΔM2-2 viruses.

## Results

### Differences in growth between the two rA2ΔM2-2 vaccine candidates

**Fig. 1.**
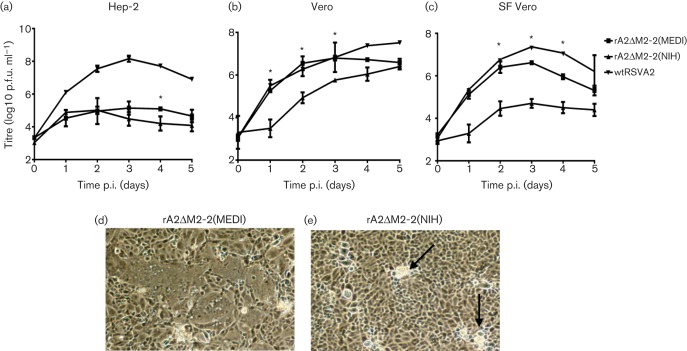
Multicycle growth of rA2ΔM2-2 viruses in three cell lines. (a–c) HEp-2 (a), Vero (b) or SF Vero (c) cells were infected at an m.o.i. of 0.1. wt RSV strain A2 (wtRSVA2) was used as a control. Cells and supernatants were harvested together at 24 h intervals and titrated by plaque assay on Vero cells. Data points represent the means±sd of two experiments. Statistically significant differences between the rA2ΔM2-2 viruses are indicated by an asterisk (*P<0.05). (d, e) Syncytium formation by rA2ΔM2-2(MEDI) (d) or rA2ΔM2-2(NIH) (e) was captured at 48 h post-infection (p.i.) at ×40 magnification using a Nikon Eclipse TS100 microscope. Arrows in (e) indicate the location of phase-bright cell clusters.

Two laboratories have previously reported the generation of rA2ΔM2-2 virus (Bermingham & Collins, 1999; Jin *et al.*, 2000). Both reported that rA2ΔM2-2 virus had reduced growth in certain cell lines, as well as attenuated growth in rodents and non-human primates (Teng *et al.*, 2000; Jin *et al.*, 2000). The multicycle growth-curve analysis of rA2ΔM2-2(MEDI) and rA2ΔM2-2(NIH) in three different cell lines is shown in Fig. 1. In HEp-2 cells, both rA2ΔM2-2 viruses grew poorly, with peak titres >100-fold lower than titres of wt RSV A2 (Fig. 1a). These results were in alignment with previous reports describing reduced growth of rA2ΔM2-2 in human cell lines such as Hep-2 (Jin *et al.*, 2000; Bermingham & Collins, 1999). Next, we compared growth in a Vero cell line obtained from ATCC as well as a SF-adapted Vero cell line used in the manufacturing of a clinical trial vaccine (Yuk *et al.*, 2006). A previous report demonstrated that growth of rA2ΔM2-2 in Vero cells was equivalent to wt RSV A2 (Jin *et al.*, 2000). In our experiments in Vero cells, rA2ΔM2-2(MEDI) had faster growth kinetics compared with rA2ΔM2-2(NIH) (Fig. 1b). On day 2 post-infection (p.i.), rA2ΔM2-2(MEDI) had a titre of 6.3 log_10_ p.f.u ml^−1^ and rA2ΔM2-2(NIH) had 4.7 log_10_ p.f.u ml^−1^, although both rA2ΔM2-2(MEDI) and rA2ΔM2-2(NIH) reached a titre of approximately 6.5 log_10_ p.f.u ml^−1^ on day 5. The difference in growth kinetics was even more evident in SF Vero cells, where rA2ΔM2-2(MEDI) had 100-fold higher titre than rA2ΔM2-2(NIH) by day 2 p.i. (Fig. 1c). In SF Vero cells, rA2ΔM2-2(MEDI) reached a peak titre of 6.6 log_10_ p.f.u ml^−1^, whilst rA2ΔM2-2(NIH) reached a peak titre of 4.6 log_10_ p.f.u ml^−1^.

In addition to the differences in growth kinetics, these viruses showed marked differences in CPE. Vero cells infected with rA2ΔM2-2(MEDI) showed large syncytia, which spread over the entire cell monolayer by 48 h p.i. (Fig. 1d). In contrast, the rA2ΔM2-2(NIH) virus produced small syncytia that were associated with phase-bright cell clusters (Fig. 1e). Although these phase-bright cell clusters increased in number over time, the rA2ΔM2-2(NIH) virus never generated the larger syncytia characterized by rA2ΔM2-2(MEDI). Similar differences in CPE were observed in the SF Vero cell line. These results showed that the rA2ΔM2-2(MEDI) virus had faster growth kinetics and generated larger syncytia in Vero cells compared with rA2ΔM2-2(NIH).

### Identification of K66E as the major genetic determinant for altered growth in Vero cells

In order to identify the genetic determinants responsible for the growth differences between these two viruses, we performed an alignment of their cDNA sequences. Although both rA2ΔM2-2(MEDI) and rA2ΔM2-2(NIH) are derived from RSV strain A2, there are differences in their genomic sequences and in the manner of the M2-2 deletion. The rA2ΔM2-2(MEDI) virus was generated by deletion of 234 nt encoding the C-terminal 78 aa of the M2-2 protein (Jin *et al.*, 2000). The rA2ΔM2-2(NIH) virus was generated by deletion of 241 nt from the same region. The results of the cDNA alignment identified 34 nt differences: four differences encoding amino acid changes in the NS2, N and F genes, 15 differences in coding regions that did not alter amino acid sequence and eight differences in the non-coding regions and the remainder in the M2-2 deletion (Table 1).

**Table 1.** Genetic differences between rA2ΔM2-2(MEDI) and rA2ΔM2-2(NIH) Numbering is based on rA2ΔM2-2(MEDI) sequence.

Nucleotide position	Genotype (MEDI → NIH)	Mutation
779	G → A	NS1 (R51K)
1209	G → A	N (A24T)
5856	A → G	F (K66E)
5962	A → C	F (Q101P)
404	C → T	
1181	G → A	
1937	G → A	
2999	A → G	
3002	A → G	
4308	A → G	
5708	T → C	
6215	C → T	silent
7214	C → T	
7611	A → T	
7612	C → A	
7701	G → C	
10280	T → C	
13399	A → C	
13666	A → C	
1099	- → C	
1138	A → G	
1139	G → C	
3094	A → G	non-coding region
5611	A → G	
5615	A → T	
5639	G → A	
7481	T → C	

The four nucleotide differences encoding amino acid changes were introduced individually into the rA2ΔM2-2(MEDI) cDNA. A fifth cDNA was generated in which the M2-2 gene deletion in rA2ΔM2-2(MEDI) was changed to mimic the analogous deletion in rA2ΔM2-2(NIH). Four rA2ΔM2-2(MEDI) viruses each carrying a single amino acid change (R51K in NS2, A24T in N, K66E in F and Q101P in F) and one carrying the M2-2 deletion of rA2ΔM2-2(NIH) were generated from these cDNAs by reverse genetics for comparison of growth kinetics and CPE.

**Fig. 2.**
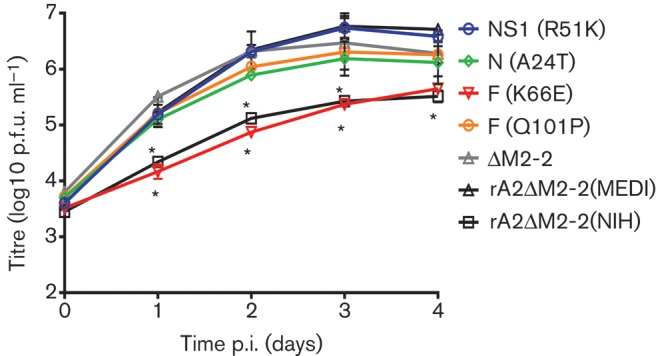
Multicycle growth of the rA2ΔM2-2(MEDI) variants. SF Vero cells in six-well plates were infected with each virus at an m.o.i. of 0.1. Cells and supernatants were harvested together at 24 h intervals and titrated by plaque assay on Vero cells. Data points represent the means±sd of two experiments. Statistically significant differences compared with rA2ΔM2-2(MEDI) are indicated by an asterisk (*P<0.001).

Both rA2ΔM2-2(NIH) and rA2ΔM2-2(MEDI)/K66E had similar growth kinetics with peak titres of 5.3 and 5.5 log_10_ p.f.u. ml^−1^, respectively (Fig. 2). The variant rA2ΔM2-2(MEDI)/K66E in Vero cells also formed the same phase-bright cell clusters seen previously with rA2ΔM2-2(NIH). In contrast, the variants harbouring R51K in NS2, A24T in N, Q101P in F and the NIH M2-2 deletion sequence all generated CPE and peak titres that were similar to rA2ΔM2-2(MEDI) (Fig. 2). These results suggest that the K66E change in the F protein is the major genetic determinant for the reduced growth and altered CPE of rA2ΔM2-2(NIH) as compared with rA2ΔM2-2(MEDI).

### A change at aa 66 in RSV F alters fusion activity

**Fig. 3.**
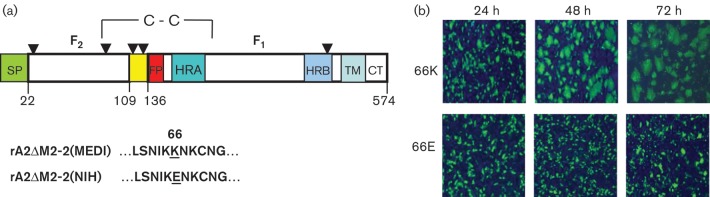
Effect of the K66E amino acid substitution on RSV F syncytium formation. (a) Linear representation of RSV F. The F_2_ fragment extends from aa 22 to 109 and the F_1_ fragment from aa 136 to 574. Potential *N*-glycosylation sites (aa 27, 70, 116, 120, 126 and 500) are denoted by arowheads. SP, signal peptide; HRA, heptad repeat A; FP, fusion peptide; HRB, heptad repeat B; TM, transmembrane region; CT, cytoplasmic tail. The SP is cleaved at aa 22, and the furin cleavage sites are aa 109 and 136. The amino acid sequence flanking K66E is shown below the diagram. (b) Vero cells were transfected with pF/66K or pF/66E. Cells were fixed at 24 h intervals and immunostained with RSV F-specific antibody in order to visualize the relative sizes of syncytia.

In order to analyse the fusion activity of the RSV F protein outside the context of virus replication, a sequence-optimized version of the RSV F gene was cloned into plasmid pCMV-Script. Transfection of Vero cells with this plasmid carrying a lysine (K) at aa 66 in the RSV F gene (pF/66K) (Fig. 3a) generated large syncytia by 72 h (Fig. 3b). In contrast, Vero cells transfected with the same plasmid carrying a glutamic acid residue (E) in the RSV F gene at aa 66 (pF/66E) (Fig. 3a) formed only small syncytia (Fig. 3b). The differences in syncytium formation observed in Vero cells transfected with pF/66K and pF/66E matched the CPE differences seen in Vero cells infected with the rA2ΔM2-2(MEDI) and rA2ΔM2-2(NIH) viruses, respectively. These results suggested that a single amino acid at position 66 in RSV F may play an important role in promoting fusion.

### A positive charge at aa 66 promotes fusogenicity

**Fig. 4.**
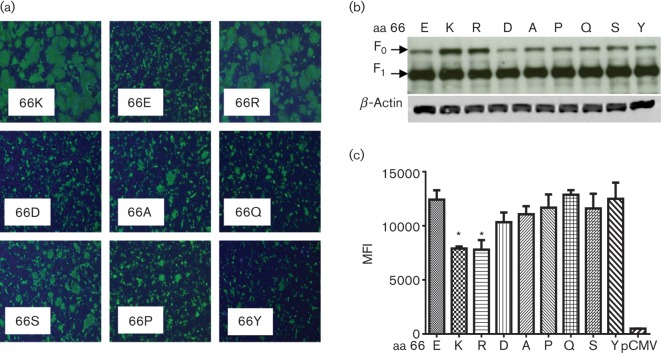
Effect of amino acid substitutions at position 66 on RSV F syncytium formation. (a) Vero cells were transfected with pF plasmids. The amino acids at position 66 are denoted within each panel. Cells were fixed at 48 h and immunostained with an RSV F-specific antibody to visualize relative sizes of syncytia. (b) Western blot of lysates from Vero cells transfected with pF plasmids. Letters above lanes denote the amino acid at position 66. The blots were probed with RSV F-specific antibody followed by β-actin-specific antibody for normalization. (c) Expression of RSV F on surface of transfected 293T cells as determined by mean fluorescence intensity (MFI). Transfections were performed in triplicate, and results that were significantly different from the MFI with an E at aa 66 are indicated with an asterisk (*P<0.005). Letters on the *x*-axis denote the amino acid at position 66. pCMV, empty pCMV-Script as a control.

To determine whether the charge at position 66 was responsible for the differences in fusion, we generated additional pCMV/RSVF plasmids carrying either a positively charged arginine (pF/66R) or a negatively charged aspartic acid (pF/66D) at position 66. Transfection experiments showed that the RSV F mutant containing 66R produced large syncytia by 48 h, whereas the RSV F mutant containing 66D produced only small syncytia (Fig. 4a). Thus, the 66K and 66R substitutions with positively charged side chains promoted fusion, whilst 66E and 66D substitutions with negatively charged side chains hindered fusion.

To further test the influence of amino acid side chain on fusion, RSV F mutants containing substitutions with uncharged side chains were similarly generated. Alanine (A) and serine (S) are commonly used in functional mutagenesis studies and carry very short side chains that are hydrophobic and neutral, respectively. Neither of these substitutions restored fusogenicity to the levels of the 66K or 66R mutants (Fig. 4a). At the opposite end of the spectrum, proline (P) and tyrosine (Y) have side chains that contain large, bulky rings that could affect secondary structure or exert steric hindrance. As expected, neither 66P nor 66Y substitution enhanced the fusogenicity of RSV F (Fig. 4a). Interestingly, a glutamine (Q) substitution at residue 66 also failed to promote fusogenicity (Fig. 4a). Glutamine is structurally similar to glutamic acid except for the charge of the side chain, with glutamine carrying a neutral side chain and glutamic acid (E) carrying a negatively charged side chain. This indicated that simple removal of the negative charge at residue 66 was not sufficient to restore the fusogenicity of the 66E mutant to the level of the 66K mutant. Taken together, these results strongly suggested that electrostatic charge interactions at position 66 in the F_2_ fragment of RSV F play a role in fusion.

The RSV F protein is initially produced as a full-length precursor (F_0_), which is cleaved by a furin-like protease to form two disulfide-linked fragments (F_1_ and F_2_) of ~50 and ~25 kDa, respectively. To confirm that the level of expression and proteolytic cleavage was equivalent among the different RSV F mutants, SDS-PAGE and Western blotting was performed on lysates of transfected Vero cells. Blots probed with motavizumab, an anti-RSV F mAb, to visualize F_0_ and F_1_ indicated that the mutants had similar levels of expression and processing at the furin cleavage site (Fig. 4b). Blots were reprobed with anti-β-actin to show equivalent amounts of protein loaded in each lane (Fig. 4b).

As different levels of RSV F on the cell surface could also have an effect on syncytium formation, we compared cell-surface expression levels of the various F mutants using flow cytometry. 293T cells were transfected with each plasmid, stained with motavizumab to detect cell-surface RSV F and subjected to FACS analysis. Surprisingly, the two constructs that caused the most cell-to-cell fusion, pF/66K and pF/66R, actually showed slightly less RSV F on the cell surface compared with cells that had been transfected with the other F plasmids (Fig. 4c). These results suggested that the larger syncytia produced by pF/66K and pF/66R were not due to a larger amount of F protein on the cell surface but were due to the ability of a positively charged residue at position 66 in F to promote fusion.

## Discussion

rA2ΔM2-2(NIH) and rA2ΔM2-2(MEDI) are two RSV vaccine candidates that are attenuated by deletion of the M2-2 ORF. Although both viruses are derived from RSV strain A2, they carry four amino acid differences and have different deletions of the M2-2 gene. In a SF-adapted Vero cell line employed for manufacturing clinical trial vaccines, rA2ΔM2-2(MEDI) grew to 100-fold higher titres than rA2ΔM2-2(NIH), with markedly larger syncytia. Genetic mapping demonstrated that an amino acid difference at position 66 of the F protein was the major determinant responsible for the observed growth and fusion differences seen between these two viruses. Moreover, syncytium-formation experiments showed that positively charged amino acids at position 66 promoted fusion, whilst negatively charged amino acids hindered fusion activity, suggesting that electrostatic charge interactions at aa 66 of the F_2_ fragment play an important role in fusion.

The K66E mutation has been described previously as part of a group of ‘HEK’ mutations identified following seven passages of a 1961 RSV A2 clinical isolate in human embryonic kidney (HEK) cells (Connors *et al.*, 1995; Whitehead *et al.*, 1998). The growth and CPE differences that we observed in both Vero and SF Vero cells were surprising, because the HEK-passaged virus from which rA2ΔM2-2(NIH) is derived was shown previously to grow well in Vero cells (Teng *et al.*, 2001; Teng & Collins, 1999). In addition, a low level of natural polymorphism at aa 66 in the F sequence has been observed in circulating wt RSV strains with no reported deleterious effect on viral fitness (Zhu et al., 2012). The results shown here underscore the complex interplay of viral and host-cell factors that can affect virus replication and fusion. Deletion of the M2-2 gene, which regulates the switch from transcription to replication, results in an imbalance that favours transcription and, in turn, viral protein expression (Bermingham & Collins, 1999). It is possible that this imbalance facilitated the identification of the K66E functional mutation described here. In addition, rA2ΔM2-2 virus has been shown to grow well in non-human cell lines such as Vero, which made it easier to identify a mutation that hindered growth as opposed to a cell line such as Hep-2 where growth of rA2ΔM2-2 virus is already significantly impaired (Jin *et al.*, 2000).

Aa 66 is located in the F_2_ fragment of the fully processed RSV F protein. Much of the published work on the function of RSV F has focused on the role of specific domains in the larger, membrane-attached F_1_ fragment, such as the fusion peptide and heptad repeats A and B (Fig. 3a). Studies on the function of specific residues within the F_2_ fragment have been limited to the role of moieties such as the cysteine (C) residue at position 69, which is involved in intermolecular disulfide bond formation, and the asparagine (N) residue at position 70, which is a potential site for *N*-glycosylation (Day *et al.*, 2006; Zimmer *et al.*, 2001). Although aa 66 is in close sequence proximity to both C69 and N70, no specific functional role has been assigned to this residue. Our syncytium-formation experiments showed that a change at aa 66 of RSV F can have a significant impact on fusion, with basic positively charged amino acids (K or R) promoting fusion and acidic negatively charged amino acids (E or D) impairing fusion. Because the differences in fusion activity seen between the F mutants cannot be explained by differences in proteolytic processing or cell-surface expression, we believe that amino acid changes at position 66 are probably having a direct effect on the fusogenicity of F.

**Fig. 5.**
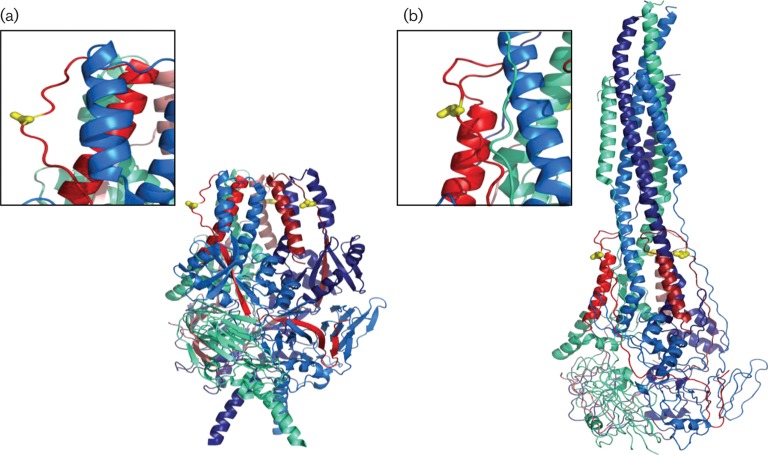
Structure of the RSV F homotrimer. The F_2_ fragment within each RSV F monomer is a different shade of red, and the F_1_ fragment within each RSV F monomer is a different shade of blue. Aa 66 is shown in yellow. (a) Pre-fusion model based on PDB 4JHW (McLellan *et al.*, 2013). (b) Post-fusion model based on PDB 3RRT (McLellan *et al.*, 2011).

The F_2_ fragment was identified previously by Schlender *et al.* (2003) as being responsible for the host-cell specificity of RSV, suggesting that it is exposed and available for direct contact with host cells during virus infection. In more recent work by McLellan *et al.* (2013), a pre-fusion structure model of RSV F was generated by co-crystallization with an antibody specific for the pre-fusion form. In this model, aa 66 is localized to the outer surface of the homotrimer near the top of the head region (Fig. 5a). The structure model of the post-fusion form also places aa 66 on the outer surface of the homotrimer (Fig. 5b). This postulated location of aa 66 allows us to propose two mechanisms by which disruption of charge could alter fusion activity.

The first hypothesis suggests that a change in charge at aa 66 alters the ability of F to bind cell-surface receptors, thereby influencing syncytium formation and spread of the virus. Nucleolin and glycosaminoglycans (GAGs) have been identified as potential cell-surface receptors for RSV virus, and there is evidence that RSV F alone can also bind GAGs (Hallak *et al.*, 2000a, b; Feldman *et al*., 2000;Martínez & Melero, 2000; Tayyari *et al.*, 2011). Because GAGs such as heparan sulfate are negatively charged, one could hypothesize that increasing the positive charge on the outer surface of the F protein enhances virus binding and, in turn, fusion. A study by Feldman *et al.* (2000) identified a putative heparin-binding domain within the F_2_ fragment that included aa 66, whilst work by Crim *et al.* (2007) using overlapping, linear peptides showed that RSV F peptides encompassing aa 66 could bind to GAGs and to Vero cells. However, binding of these peptides to Vero cells failed to inhibit subsequent binding of RSV (Crim *et al.*, 2007). In addition, recently published experiments by McLellan *et al.* (2013) demonstrated that binding of a mAb to a pre-fusion epitope that included aa 66 had no effect on viral attachment, suggesting that this residue does not play an important role in binding of RSV to host cells.

The second hypothesis proposes that the charge of the amino acid at position 66 in RSV F affects local intra- and/or intermolecular electrostatic interactions and, in turn, the ability to transition from pre- to post-fusion conformation. Gardner & Dutch (2007) identified a region spanning the C terminus of the F_2_ fragment that is relatively well conserved in a variety of paramyxoviruses and found that mutations in this conserved region affected fusogenicity. Chang *et al.* (2012) also demonstrated the importance of charged residues in the F_2_ fragment for electrostatic interactions and the overall stability of the human metapneumovirus F protein. In both the pre-fusion (Fig. 5a) and post-fusion (Fig. 5b) models of RSV F, aa 66 is located on an exposed loop that is not in close proximity to known functional domains (McLellan *et al.*, 2011, 2013). Our analysis of these structure models failed to identify any potential side-chain interactions between aa 66 and neighbouring residues, making speculation on the effect of the K66E mutation difficult. The overall charge distribution in the region surrounding aa 66 is highly positive; therefore, insertion of a negatively charged residue could stabilize the pre-fusion structure and, in turn, increase the threshold for triggering. Alternatively, the slight inward shift of the loop containing residue 66 between the pre- and post-fusion structure models raises the possibility that the side chain of aa 66 is interacting with other unknown residues during the massive structural rearrangement that constitutes fusion. Our work has demonstrated that a change in charge at aa 66 can have a significant impact on the fusogenicity of RSV F; however, elucidation of structural intermediates of fusion may be required in order to understand fully the precise role of this residue.

## Methods

### 

#### Cell lines and virus.

Vero cells (ATCC) were maintained in minimal essential medium (MEM; Gibco) supplemented with 5 % heat-inactivated FBS (Hyclone), 2 mM l-glutamine (Gibco), and 100 U penicillin ml^−1^ and 100 µg streptomycin ml^−1^ (BioWhittaker). SF-adapted Vero cells have been described previously (Yuk *et al.*, 2006) and were maintained in OptiPro SFM (Gibco) supplemented with 2 mM l-glutamine, 100 U penicillin ml^−1^ and 100 µg streptomycin ml^−1^. 293T cells (ATCC) were maintained in Dulbecco’s MEM (Gibco) supplemented with 10 % heat-inactivated FBS, 2 mM l-glutamine, 100 penicillin U ml^−1^ and 100 µg streptomycin ml^−1^. BSR/T7 cells (kindly provided by K. K. Conzelmann, Ludwig-Maximilians-Universitat Munich) were maintained in Glasgow MEM (Gibco) supplemented with 10 % heat-inactivated FBS, 2 % tryptone-phosphate broth (Sigma) and 100 µg geniticin (Gibco) ml^−1^. All cell lines were cultured at 37 °C, 5 % CO_2_. wt RSV strain A2 (ATCC) was passaged in Vero cells.

To propagate virus, Vero cells were infected at an m.o.i. of 0.01 in Opti-MEM I (Gibco). When the CPE covered 70–80 % of the monolayer, cells and supernatant were harvested together. Cryo-preservative (10×: 2.18 M sucrose, 0.038 M KH_2_PO_4_, 0.072 M K_2_HPO_4_ at pH 7.1) was added to a final concentration of 1×, and the samples were vortexed, aliquotted and flash frozen in a dry ice/ethanol bath for storage at −70 °C.

#### Plasmids.

Two subclones spanning the areas of interest were used to make nucleotide changes in the full-length rA2ΔM2-2(MEDI) cDNA. The subclones were derived from plasmid pA2ΔM2-2 as described previously (Jin *et al.*, 2000). The first subclone was generated by digesting pA2ΔM2-2 with *Kpn*I and *Xho*I (NEB) and ligating the 4482 bp fragment into plasmid pCITE-2a (Agilent). The resulting clone was designated pCITERSV/K-X, and comprised nt 1–4482 of the rA2ΔM2-2(MEDI) cDNA. The second subclone was generated by digesting plasmid pA2ΔM2-2 with *Xho*I and *Bam*HI (NEB) and ligating the 3785 bp fragment into plasmid pCR-2.1 (Life Technologies). The resulting subclone was designated pCR2.1RSVΔM2-2/X-B and comprised nt 4482–8267 of the rA2ΔM2-2(MEDI) genome. Nucleotide changes in each subclone were made using a QuikChange Site-Directed Mutagenesis kit, following the manufacturer’s instructions (Agilent). Nucleotide changes were confirmed by sequencing, and the fragments were inserted back into the full-length pA2ΔM2-2 cDNA using the same paired restriction enzymes described above for each subclone. For transfection experiments requiring expression of the full-length RSVA2 F protein, the 1725 bp sequence of the RSV F ORF was optimized at MedImmune and the DNA synthesized by DNA2.0. The ORF was amplified by PCR and cloned into plasmid pCMV-Script (Agilent). This plasmid was designated pCMV/RSVF (pF). Nucleotide changes in the RSV F sequence were made using a QuikChange Site-Directed Mutagenesis kit.

#### Rescue of recombinant rRSVA2ΔM2-2 virus.

Six-well plates of subconfluent BSRT7 cells were co-transfected with plasmid encoding the full-length cDNA as well as helper plasmids encoding the RSV A2 N, phosphoprotein (P), M2-1 and large (L) genes under the control of the T7 promoter. Briefly, 4 µg full-length cDNA was mixed with 0.4 µg pCITE/RSV N, 0.4 µg pCITE/RSV P, 0.3 µg pCITE/RSV L and 0.2 µg pCITE/RSV M2-1, and 8 µl Lipofectamine 2000 (Life Technologies) in a final volume of 0.2 ml Opti-MEM I. BSRT7 cells were washed and 0.5 ml Opti-MEM I was added followed by 0.2 ml transfection mix. The plates were incubated overnight at 35 °C. The following day, the transfection mix was removed and replaced with 2 ml Opti-MEM I. After 5 days’ incubation at 35 °C in a 5 % CO_2_ incubator, the cells and supernatant were harvested together as described above. Rescued virus was amplified by two to three passages in Vero cells. Viral titres were determined by plaque assay.

The sequence of each recombinant virus was confirmed by reverse transcription (RT)-polymerase chain reaction (PCR). Briefly, the viral RNA was isolated using a Qiamp Viral RNA Mini kit (Qiagen). RT-PCR was performed using a OneStep RT-PCR kit (Qiagen) and oligonucleotide primers that generated overlapping PCR products covering the entire genome. Gel-extracted PCR products (Qiagen) were sent to Sequetech Inc. for sequencing.

#### Plaque assay.

Virus stocks were serially diluted and 0.5 ml of each dilution was used to infect one well of a six-well plate containing subconfluent Vero cells. After 1 h of rocking at room temperature, the virus was aspirated and wells were overlaid with a 1 : 1 mixture of 2 % methylcellulose and 2× L-15/EMEM (SAFC) supplemented with 2 % heat-inactivated FBS, 4 mM l-glutamine, 200 U penicillin ml^−1^ and 200 µg streptomycin ml^−1^. Plates were incubated at 35 °C in a 5 % CO_2_ incubator. After 5–6 days’ incubation, the overlay was removed by aspiration, the plates were fixed in methanol and the fixed cells were immunostained using polyclonal anti-RSV antibody (Millipore) diluted 1 : 1000 in 5 % powdered milk (w/v) in PBS, followed by HRP-conjugated rabbit anti-goat antibody (Dako). Plaques were visualized with 3-amino-9-ethylcarbazole (Dako). Virus titre is reported as p.f.u. ml^−1^.

#### Multicycle growth analysis of recombinant rRSVA2ΔM2-2 virus.

Six-well plates of subconfluent Vero cells were infected at m.o.i. of 0.1 in 0.5 ml Opti-MEM I per well. Plates were rocked at room temperature for 1 h to facilitate virus absorption and washed once with Opti-MEM I, followed by the addition of 2 ml per well of fresh Opti-MEM I. Plates were incubated at 35 °C with 5 % CO_2_, and virus was harvested at 24 h intervals as described above. Samples were stored at −70 °C and titrated by plaque assay as described above.

#### Syncytium-formation assay.

Subconfluent Vero cells in six-well plates were transfected overnight with 1 µg per well of plasmid pF or its derivatives. Briefly, transfection mix was generated by mixing 4 µl Lipofectamine 2000 per 1 µg plasmid DNA in a final volume of 0.2 ml Opti-MEM I. Cells were washed once and 0.5 ml Opti-MEM I was added per well, followed by 0.2 ml transfection mix per well. After overnight incubation at 37 °C with 5 % CO_2_, the plates were washed and 2 ml per well of Opti-MEM I was added before continued incubation at 37 °C. Syncytium formation was examined at various time points post-transfection, and images were captured using a Nikon Eclipse TS100 microscope.

#### Western blotting.

Six-well plates of Vero cells were transfected as described above. At 48 h post-transfection, cell lysates were harvested by aspirating the medium, washing the well with PBS and adding 0.3 ml Laemmli buffer plus β-mercaptoethanol directly to each well. Before loading onto 12 % Tris/glycine SDS-PAGE gels, lysates were incubated at 95 °C for 10 min. Gels were blotted onto PVDF membranes (Life Technologies) and probed with motavizumab (0.1 µg ml^−1^), a highly potent mAb against RSV F (Wu *et al.*, 2007). This step was followed by incubation with HRP-conjugated anti-human secondary antibody (Dako) and electrochemiluminescent (ECL) detection using Supersignal Dura West ECL substrate (Pierce) and an ImageQuant LAS4000 imager (GE Healthcare). Blots were then rinsed with PBS and reprobed with an anti-chicken β-actin mAb (Millipore) to normalize for protein loading. This was followed by HRP-conjugated anti-mouse secondary antibody (Dako) and ECL detection as described above.

#### Immunofluorescence.

Vero cells were seeded to 90 % confluency in 12-well plates containing sterile glass coverslips. Transfections were performed as described above but scaled down for 12-well plates. At 48 h post-transfection, cells were fixed with 4 % paraformaldehyde in PBS for 20 min at room temperature. Plates were blocked with PBS containing 1 % BSA for 1 h at 37 °C followed by incubation with motavizumab (0.5 µg ml^−1^ in PBS containing 1 % BSA and 0.1 % saponin) for 1 h at 37 °C. Plates were washed with PBS/Tween 20 and incubated with Alexa Fluor 488-conjugated goat anti-human IgG (Life Technologies; 1 µg ml^−1^ in PBS containing 1 % BSA and 0.1 % saponin). After 1 h at 37 °C, the plates were washed with PBS/Tween 20. Coverslips were inverted and mounted on glass slides using Vectashield mounting medium with DAPI (Vector Laboratories). Images were captured at ×4 magnification using a Nikon Eclipse 80i microscope with a CoolSnapES2 camera and Simple PCI6 software.

#### Flow cytometry.

To assess cell-surface expression of RSV F, 293T cells were transfected as described above. At 20 h post-transfection, cells were stained for FACS analysis using motavizumab (1 µg ml^−1^) followed by Alexa Fluor 488-conjugated goat anti-human IgG antibody (1 µg ml^−1^), each diluted in FACS Stain Buffer (BD Pharmingen). Cells were fixed with Cytofix Fixation Buffer (BD Pharmingen) and analysed on an LSR-II flow cytometer (BD Biosciences). Mean fluorescence intensity was determined using FACSDiva software.

#### Statistical analysis.

Growth-curve data were analysed by *t*-tests using a mixed-effect model with correlated error structure. FACS data were also analysed by *t*-tests using a mixed-effect model with heterogeneous group variability.
